# Astrovascular decoupling in awake 5×FAD mice is associated with reduced astrocytic calcium

**DOI:** 10.1002/alz.70564

**Published:** 2025-08-13

**Authors:** Ruei‐Lung Lin, Sophiya L. Sims, Nicholas A. Wright, Léopoldine B. Galopin, Blaine E. Weiss, Susan D. Kraner, Jacquelyn E. Rhinehart, Ting‐Hsuan Lu, Pradoldej Sompol, Christopher M. Norris, Olivier Thibault

**Affiliations:** ^1^ Department of Pharmacology and Nutritional Sciences University of Kentucky Lexington Kentucky USA; ^2^ Sanders‐Brown Center on Aging University of Kentucky Lexington Kentucky USA

**Keywords:** aging, Alzheimer's disease, ambulation, astrocyte networks, Ca^2+^, vasoreactivity, wavelets

## Abstract

**INTRODUCTION:**

Evidence points to dysregulated Ca^2+^ in neurons and astrocytes in models of amyloidosis. While most of these data were obtained in vitro or in vivo under anesthesia, less work has investigated these variables in awake ambulating mice.

**METHODS:**

Astrocytic Ca^2+^ fluctuations (GCaMP8f) were imaged concomitantly with vasoreactivity in S1 on a two‐photon microscope during rest and ambulation. Single‐cell resolution variables were extracted using continuous wavelet transform and traditional ΔF/F measures.

**RESULTS:**

Along with increases in amyloid beta (Aβ) accumulation, we found significant reductions in measures of astrocyte functional connectivity, pairwise correlations, and network synchronicity in older 5×FAD mice, with greater decreases in females. Results align with altered gait and reduced astrovascular coupling.

**DISCUSSION:**

The results provided here are novel and demonstrate that age and sex are major risk factors for AD; however, central astrovascular dysregulations appear to exist in response to reduced, rather than elevated astrocyte Ca^2+^ transients.

**Highlights:**

- Gait analyses reveal older 5×FAD animals display shorter stride length while ambulating.
- While resting astrocytic calcium activity is increased with age, sex, and genotypes, these results do not explain changes in astrovascular coupling.
- Only small changes in astrocyte calcium transients and vessel morphology are seen, while the astrocyte‐to‐vessel correlations (astrovascular coupling) are significantly reduced.
- Astrocyte network analysis reveals significant reductions in measures of astrocytic communication and activity which are associated with reduced functional vasoreactivity in awake ambulating animals.

## 1 BACKGROUND

Alzheimer's disease (AD) and related dementias (ADRD) are devastating conditions with both peripheral and central consequences that ultimately contribute to cognitive and memory decline, gait dysregulation, and sometimes lead to depression. Despite deep scientific scrutiny, only three therapeutic approaches currently exist: acetylcholinesterase (AchE) inhibitors, uncompetitive N‐methyl‐D‐aspartate (NMDA) receptor blockers, and anti‐amyloid beta (Aβ) immunotherapy. Most molecular targets associated with these compounds interact with neuronal or astrocytic Ca^2+^ signaling, which is known to be disrupted during aging and neurodegenerative disease [Alzheimer's Association Calcium Hypothesis Workgroup,^1^].

Using electrophysiology and imaging techniques, prior work has shown significant genotype differences on measures of neuronal and astrocytic Ca^2+^ responses across animal models that recapitulate Alzheimer's pathologies. Previous work points to hyperactivity and hyperexcitability in both cell types^2^, ^3^, ^4^, ^5^, ^6^, ^7^, ^8^, ^9^, ^10^, ^11^, ^12^, ^13^ with these initial studies being limited to work in anesthetized animals, *ex‐vivo* brain slices, or primary cultures. As two‐photon (2P) microscopy has become more accessible, investigations are now conducted in vivo and on awake mice. Several labs have reported on dysregulated neuronal Ca^2+^ transients in models of amyloidosis in awake and/or anesthetized conditions, reflecting on elevations in the number of bursting or hyperactive neurons.^5^, ^8^, ^9^, ^13^, ^14^ Thus, a hyperactivity phenotype appears to persist across techniques, models of AD, and brain regions investigated.

Similarly, the hyperactive astrocytic Ca^2+^ phenotype is generally represented with increases in resting Ca^2+^ levels and transients, spontaneous activity, and/or number of active cells.^15^, ^16^, ^17^, ^18^, ^19^, ^20^, ^21^, ^22^ It should be noted; however, that evidence also points to reductions in some of the Ca^2+^ measures taken in astrocytic substructures. Indeed, others have suggested that astrocytic Ca^2+^ levels may not be linked to functional hyperemia as initially hypothesized.^23^, ^24^, ^25^, ^26^ Further, decreased cellular Ca^2+^ signaling or sensory‐evoked responses were found in the APP NL‐F model,^13^ the Tg‐ArcSwe mouse,^27^ the APP/PS1,^19^, ^28^ and the 5×FAD mouse.^29^, ^30^ These data suggest that Ca^2+^ dysregulation may not be a unidirectional phenomenon. Finally, little work has directly measured network dynamics, overall cellular connectivity, or synchronicity concomitantly with vascular function in in vigilo, awake, and behaving settings.

Astrocytes unsheathe much of the cerebrovasculature, and astrocyte Ca^2+^ signaling has been mechanistically linked to vasoreactivity; thus, preserving the fidelity of the astro‐neurovascular unit appears to be essential for maintaining brain function^31^. The 2P, positron emission tomography (PET), and ex vivo imaging techniques applied to a variety of anesthetized AD mouse models reveal reduced astro‐neurovascular coupling, vessel density, and abnormal vascular responses/tone and compliance, as well as increases in microhemorrhages.^16^, ^18^, ^32^, ^33^, ^34^ Recent work in awake mice showed that diet‐induced hyperhomocysteinemia increases astrocytic Ca^2+^ transients, reduces vasodilation and red blood cell velocity, and increases vessel leakiness.^35^ The ability to evaluate vasoreactivity concomitantly with astrocytic activity in awake mice is critical for understanding the mechanistic impact of either element in the context of AD or AD‐like pathology.

**RESEARCH IN CONTEXT**

1. **Systematic review**: We reviewed the literature for prior published work conducted in animal models of amyloidosis focusing on measures of calcium dysregulation in astrocytes. This type of brain cell engages with vascular elements, and is known to participate in astrovascular coupling, the local functional changes that alter cerebral blood flow during activation. Prior work emphasizes hyperactivity in astrocytic calcium networks, and suggests this could drive the decoupling between astrocytes and vasoreactivity.
2. **Interpretation**: We investigated astrocyte calcium changes at rest and during ambulation and tested for the impact on vascular function across eight groups of animals separated by age, sex, and genotype in a model of amyloidosis and its controls. Our work shows that calcium‐dependent communication measures in cortical astrocytes of mice during ambulation are reduced in the older 5×FAD compared to controls. We suggest these reductions in astrocyte network activity may be responsible for astrovascular decoupling. This study also shows that cortical astrocyte networks in aged 5×FAD female animals were the most impacted. Surprisingly, very few alterations in vessel morphology or reactivity were seen, again suggesting upstream astrocytic control, perhaps mediated by reduced communication, is perturbed in this model.
3. **Future directions**: This work did not test a specific hypothesis based on the mechanism by which reductions in astrocytic calcium variables could mediate the decoupling with vasoreactivity. We identified large sex and genotype alterations in the astrocytic calcium network properties, and future studies should test whether these are dependent on specific astrocytic signaling (i.e., NO, NE, PGE2, AA, ATP).

Here, for the first time, we collected concurrent 2P measures of vasoreactivity and astrocytic Ca^2+^ signaling in freely ambulating 5×FAD mice at early and late stages of plaque deposition. Using GCaMP8f signals, we investigated astrocyte networks and astrovascular reactivity changes with age, sex, and genotype. Finally, by focusing on somatosensory cortex (S1) imaging of astrocyte Ca^2+^ activity and vasoreactivity at rest and during ambulation, we align our results with clinical evidence showing dysregulation in ambulation, speed, and gait in AD and ADRD.^36^, ^37^, ^38^

## 2 METHODS

### 2.1 Breeding

Because our lab is investigating insulin receptor knockdown in astrocytes, we have crossbred the 5×FAD mouse with the homozygote insulin receptor floxed mouse (gift from Dr. Greg Graf, University of Kentucky). The 5×FAD mouse model was used as it is characterized by several mutations seen in the clinic, reproducing validated features of AD pathology, including neuronal cell loss, Aβ deposition, inflammation, synaptic loss, and astrogliosis. Briefly, to ensure a steady population of homozygote IR*Lox* × 5×FAD mice of both sexes, we initially bred the homozygote female IR*Lox* mice with 5×FAD males on a C57BL/6J background (Jackson Lab stock #34848; B6.Cg‐Tg(APPSwFlLon, PSEN1*M146L*L286V)6799Vas/Mmjax). The first generation of offsprings (IRfl+/5×FAD+) were then crossed with mice that were IRfl+/5×FAD‐, to provide IRflfl/5×FAD+ (IR double floxed/5×FAD+) and IRflfl/5×FAD‐ (IR double floxed/5×FAD‐) mice. The second generation of offspring were bred, providing the third generation consisting of 50% IRflfl/5×FAD+ and 50% IRflfl/5×FAD‐. For simplicity, we refer here to these animals as either controls (IRflfl/5×FAD‐) or 5xFAD (IRflfl/5×FAD+). Note that we did not use Cre‐recombinase to alter insulin receptor levels in astrocytes in the current study; however, all animals used for imaging received a mixture of GCaMP8f and Luciferase (see below). All animals used were housed in our animal facility under a reverse light cycle (OFF: 8AM; ON: 8 PM) 1 week prior to imaging. Work presented here strictly adheres to our Institutional Animal Care and Use Committee and was approved (IACUC# 2022‐4031).

### 2.2 Gait characterization

**FIGURE 1:**
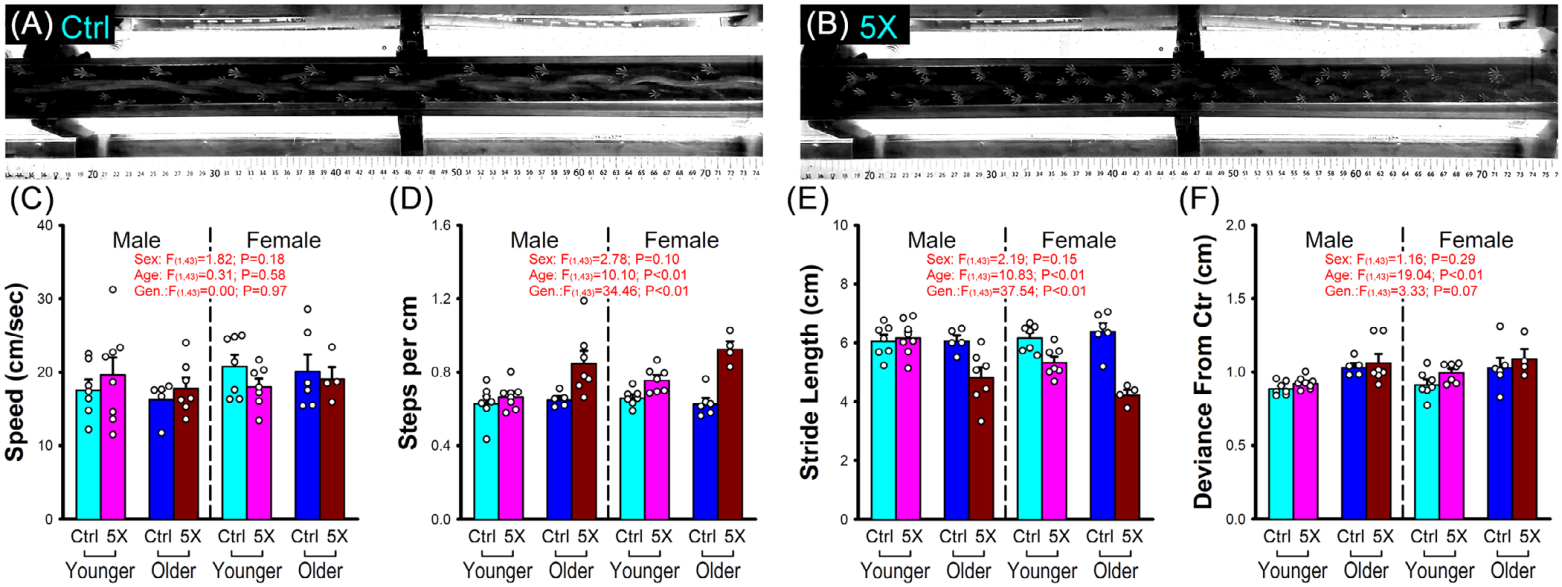
Gait performance in younger and older mice across genotype and sex. (A, B) Representative example of paw placements in older control (Ctrl) and 5×FAD mice (5×) with obvious increases in step numbers in the 5×FAD animals. Outcome measures include speed (C) (cm per sec (cm/s), steps per cm (D), stride length (E), and deviance from center (F). The measurements are obtained in younger Ctrl males (*n* = 7), 5×FAD males (*n* = 8), Ctrl females (*n* = 7), 5×FAD females (*n* = 7), older Ctrl males (*n* = 5), 5×FAD males (*n* = 7), Ctrl females (*n* = 6), and 5×FAD females (*n* = 4) during ambulation. A main effect of aging was noted for measures of steps per cm (D), stride length (E), and deviance from center (F). We also report a main effect of genotype (Gen.) for measures of steps per cm (D) and stride length (E). (C) No main effects were observed for speed. Error bars represent standard error of the mean.

A subset of the animals included in the current study were behaviorally characterized while ambulating on a three‐plane visualization walking task equipped with a 4K camera (BRIO 4K camera [25 frames per second], Logitech, Lausanne, Switzerland) and designed to extract ambulation variables that reflect relevant clinical outcome measures. These include measures of speed (cm/s), number of steps taken per cm, stride lengths (for each paw, averaging the distance between each placement across steps), and deviance from center (average of the deviation of each paw placement from the center of the corridor across multiple steps). Paw placement measures (i.e., contact points with floor, see Figure 1A,B) were obtained from videos of animals ambulating at least 4 consecutive steps (Image J (v1.54f)).

### 2.3 Immunohistochemistry

Following ice‐cold saline cardiac perfusions, brains were post‐fixed in 4% paraformaldehyde (PFA) and phosphate‐buffered saline (PBS) for 24 h, followed by a switch to a 30% sucrose solution in PBS, and an antifreeze solution (15% sucrose/30% ethylene glycol in PBS) for storage (−20°C) until sectioning and antibody labelling. Tissue sections (40 µm) were obtained anterior to the center of S1 using a cryostat (Thermo Fisher, Waltham, MA). Sections were washed three times (10 min each) with tris‐buffered saline (TBS) supplemented with 0.1% Triton X‐100 (TBS/Triton X‐100). Sections were then placed in blocking buffer for 30 min (TBS/Triton S‐100, 5% bovine serum albumin, and 3% goat serum). Free‐floating sections were then exposed to primary antibodies (rabbit anti‐β amyloid 1‐42, 1:1000, Abcam 201060; and rat anti‐glial fibrillary acidic protein (GFAP) 1:200, Invitrogen 13‐0300) for 2 h at room temperature or overnight at 4°C on a shaker. All antibody solutions were made with the blocking solution. Sections were then washed three times with TBS/Triton X‐100 and blocked for 30 min for a second time to reduce non‐specific binding of the secondary antibody. Secondary antibodies (Alexa Fluor 594, 1:200, Invitrogen A11072; and Alexa Fluor 488, 1:200, Invitrogen A21210) were sequentially introduced each for a 2 h incubation period at room temperature or overnight at 4°C on a shaker and washed three times after the first incubation (TBS/Triton X‐100). Sections were then exposed to 4′,6‐diamidino‐2‐phenylindole (DAPI, Thermo Scientific 62248) in TBS/Triton X‐100 and washed twice (TBS/Triton X‐100). Section mounted to slides, and cover‐slipped.

### 2.4 Plaque load and astrocyte density quantification

**FIGURE 2:**
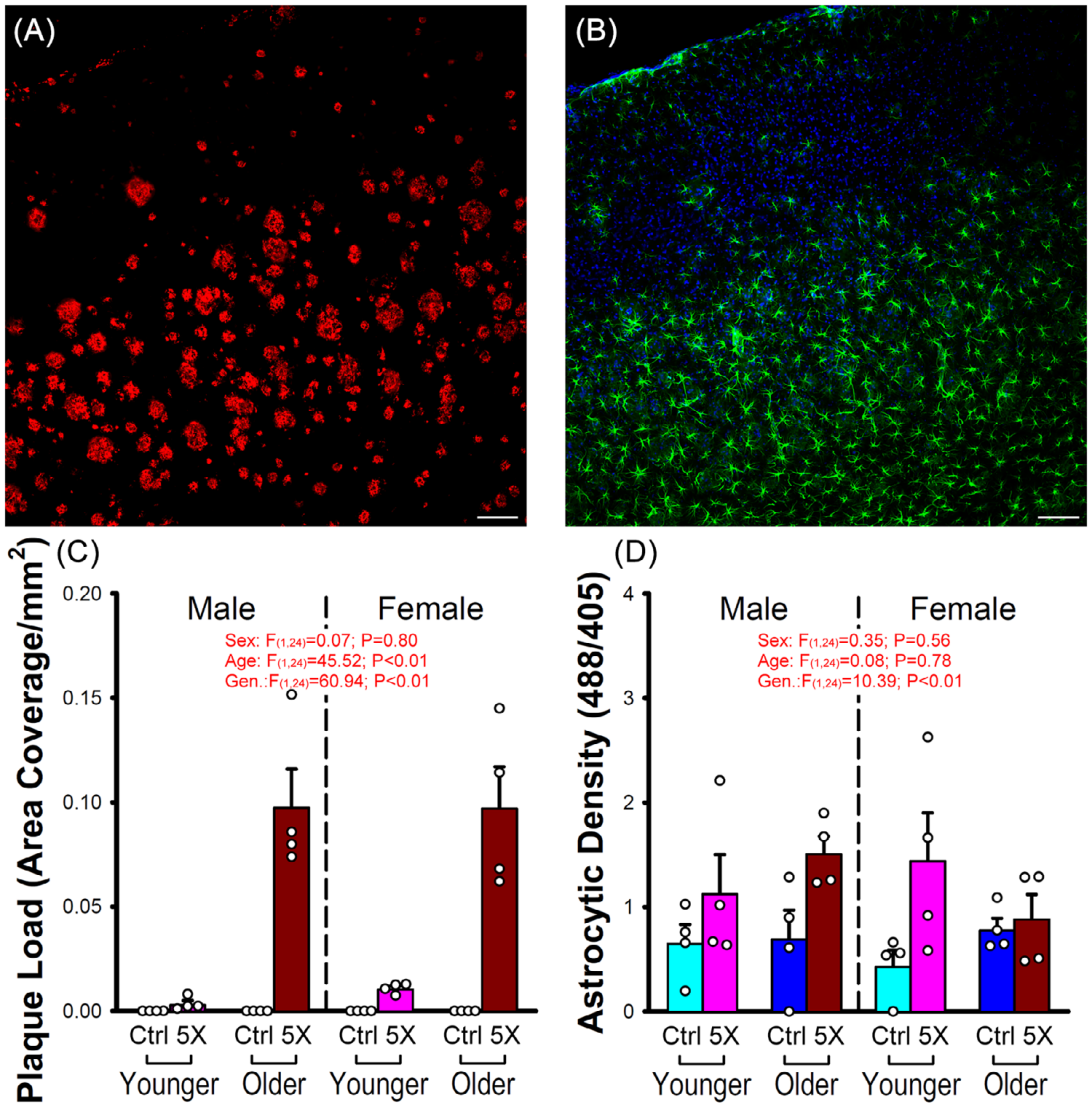
Amyloid plaque and reactive astrocyte density increase in 5×FAD. (A) Anti‐amyloid‐β immunoreactivity shows plaque accumulation in an older 5×FAD animal (red), as well as (B) anti‐glial fibrillary acidic protein (GFAP) labeling astrocytes (green), overlapped with DAPI (4′,6‐diamidino‐2‐phenylindole) nuclear staining (blue). Scale bar is 100 µm. (C) Quantitative analysis reveals main effects of age and genotype on amyloid‐β plaque load (three‐way analysis of variance [ANOVA]). (D) A main effect of genotype (Gen.) was observed showing increased astrocytic density (area covered), based on GFAP (488 nm) fluorescence intensity normalized to DAPI (405 nm). Error bars represent standard error of the mean.

All sections were visualized using the same laser settings (DAPI, 405 nm; GFAP, 488 nm; beta amyloid, 561 nm) on the stage of an A1R confocal microscope (Nikon) equipped with a 10X objective. Maximum intensity Z‐stacks were created using NIS‐Elements to quantify plaque load using a minimum aggregate size filter of 5 µm and calculating positively immunostained areas in a region covering all layers of cortex (area covered, see Figure 2). For measures of astrocyte density, the total GFAP signal (green) was normalized to the total DAPI signal (blue) in the same region. Sections were imaged in the opposite hemisphere of the injection site to lower the potential impact of surgery‐related inflammation.

### 2.5 Animal surgery and 2P imaging

A total of 96 mice underwent a craniotomy surgery (randomly completed on either side of the brain), and 64 provided bright cellular GCaMP signals, of these, 57 displayed signal changes during ambulation in cellular elements with obvious astrocyte morphology. Here, we present data from 30 younger (3–4 months old, nine male and six female controls, and nine male and six female 5×FAD) and 27 older (8–10 months old, six male and seven female controls, and eight male and six female 5×FAD) mice (see Table 1). We refer to the 3–4 months old animals as “younger” and the 8–10 months old animals as “older” to highlight statistical analyses on main effects (e.g., aging) but recognize that the animals are not truly aged (e.g., 22–36 months). Briefly, animals were anesthetized with vaporized isoflurane and a circular cranial window (3 mm in diameter) was created above the S1 region between the hind and forepaw regions (AP: −0.5 mm, ML: ± 1.62 mm) and received a mixture of GCaMP8f (AAV5‐Gfa104‐GCaMP8f; 2.22–4.85 × 10^13 GC/mL; 0.25 µL) and luciferase (AAV5‐Gfa104‐CI‐Luc.SV40; 7.14 × 10^13 GC/mL; 0.75 µL) at a depth of −0.4 mm. A 4‐mm round glass coverslip and a head plate (Neurotar model 5; Helsinki, Finland) were then sealed and mounted using light‐curing dental cement (GDT, Beer Sheva, Israel) over a thin layer of bonding agent (VivaPen, Ivoclar, Buffalo, NY). Four weeks post‐surgery, animals were acclimated to head fixing (∼2 min held by hand), then on the next day, to the Neurotar environment (Large Mobile HomeCage, Neurotar) for 2 min. During mounting, animals were briefly anesthetized with isoflurane (∼3 min) and received a retro‐orbital rhodamine dextran injection (5% w/v in sterile saline, 50 µL) to visualize blood vessels. Imaging sessions were conducted on a 2P microscope (∼30 Hz). A Scientifica Hyperscope (Uckfield, England) equipped with a water immersion 16× objective (NA = 0.8; WD = 3.0 mm; Nikon; Tokyo, Japan), two GaAsP detectors mounted inside an extra‐large collection optics detection chamber (Scientifica, MDUXL), custom sized primary dichroic and infrared blocking filters, a scanhead with two galvos, one resonant scanning mirrors, and an A/D board (National Instruments, Austin, TX) was use while communicating with ScanImage (MBF Bioscience, Williston, VT) to output a signed 16 bit image. An InSight X3 (Spectra Physics, Milpitas, CA) femtosecond‐pulsed tunable laser (930 nm excitation) was used for all light excitation. The astrocytic GCaMP8 intensity (2P green channel), vasoreactivity (2P red channel), and the animal's ambulation velocity were recorded simultaneously for each imaging session under freely moving conditions on the Neurotar environment (∼5.5 min). If an animal did not ambulate during the first session, a second session was initiated. We used a single field of view (FOV) randomly obtained (between fore and hind limb cortices) from each animal.

**TABLE 1:** Number of subjects per group.

Group #	Male				Female			
	Younger		Older		Younger		Older	
	Ctrl	5×	Ctrl	5×	Ctrl	5×	Ctrl	5×
Animal (FOV)	9	9	6	8	6	6	7	6
Astrocyte (aROI)	420	427	369	475	314	241	413	331
Vessel (vROI)	303	283	263	332	233	115	273	251

Abbreviations: aROI = astrocytic region of interest; FOV = field of view; vROI = vascular region of interest.

### 2.6 Imaging data processing and extraction

**FIGURE 3:**
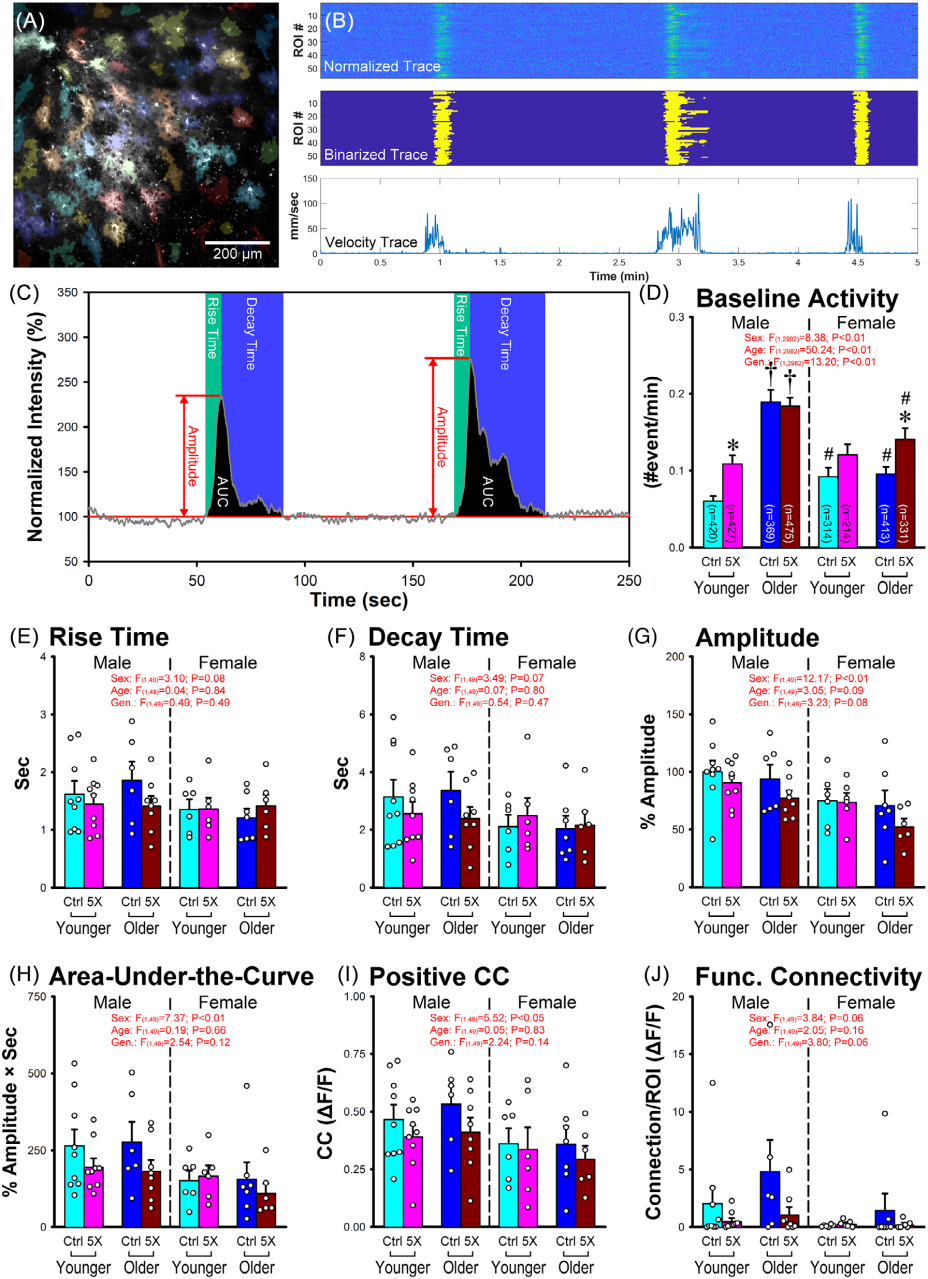
Astrocytic Ca^2+^ transients and (ΔF/F)‐based network properties across sex, age, and genotype. (A–C) Individual astrocytic regions of interest (aROIs) were identified (A), binarized (B), and variables were extracted (C). (D) Baseline activity measured across ∼3000 aROIs showed a significant increase with age and genotype, as well as a decrease in females. (E–H) Astrocytic Ca^2+^ wave properties were extracted to measure rise time (E) decay time (F), amplitude (G), and area‐under‐the‐curve (AUC). (H) Main effect of sex revealed reductions in amplitude and AUC in females. Positive correlation coefficients (CC) (I) and functional connectivity (connection/ROI) (J) were also analyzed, and again, decreases were found in CC in females. * Indicates significant difference from corresponding control (Ctrl); † indicates significant difference from the younger group; # indicates significant difference from the corresponding male group. Error bars represent standard error of the mean.

**FIGURE 4:**
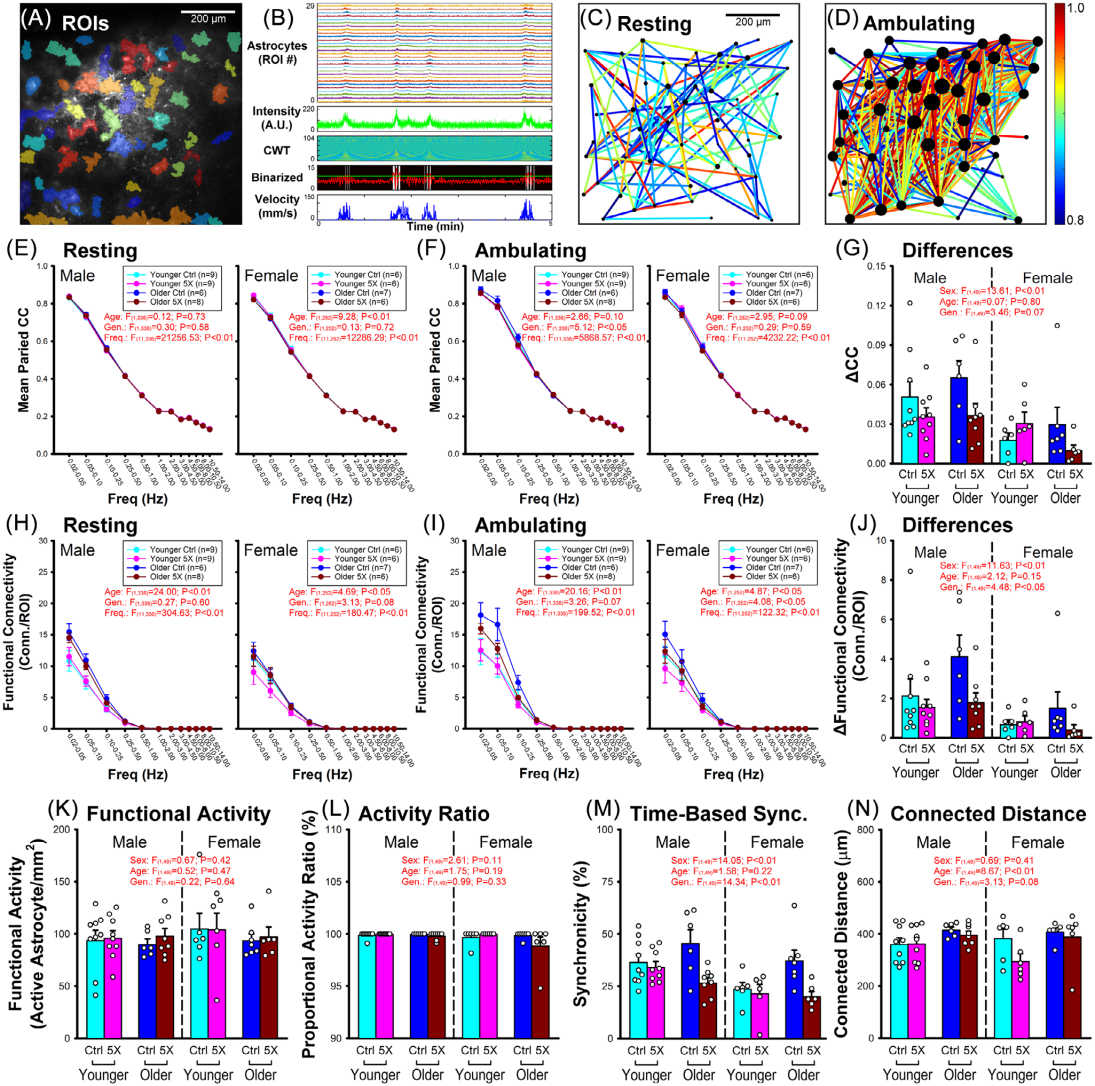
Continuous wavelet transform (CWT) analysis of astrocytic regions of interest (aROIs) to extract network properties across sex, age, and genotype. (A) Representative aROIs. (B) Individual Ca^2+^ signals (top 2 panels) during rest and ambulation underwent a CWT extraction and binarization (arbitrary unit; A.U.). (C, D) Functional connectivity across all aROIs in the FOV. Line colors represent correlation coefficient (CC) values between each astrocyte pair, while the size of the circles indicates number of connections for each aROI (color bar, right). Correlograms show increased activity, correlation, and connectivity during ambulation compared to rest. (E–G) CC analysis across frequencies (0.02–14 Hz) at rest (E) and during ambulation (F). A main effect of sex was noted in the ΔCC (difference between ambulating and resting; G). (H–J) Functional connectivity per aROI derived from CCs > 0.8 (connections/ROI), at rest (H) and during ambulation (I). Main effects of sex and genotype (Gen.) were noted in functional connectivity with ambulation (J). (K, L) No main effect was noted in functional activity (K) and activity ratio (L). (M) Main effects of sex and genotype were noted in time‐based synchronicity (Sync.). (N) A main effect of age was noted in connected distance. Error bars represent standard error of the mean.

Signal processing and data extraction were accomplished using in‐house MATLAB routines similar to those used in our previous work.^39^, ^40^ Prior to implementing the processing pipelines described below, astrocytic and vascular image stacks were imported as cubes (X, Y, and time) and corrected for movement using a jitter correction algorithm. Astrocytic regions of interest (aROIs) were defined using the average of the image cube across time (Z‐direction) to produce a single image, representing that FOV. A Gaussian blurriness (sigma = 0.8), a two‐step adaptive thresholding (both step sensitivities = 0.4) and a size filter (> 256 µm^2^) were then implemented to identify single aROIs. Signal intensities were extracted from each aROI (Figures 3A and 4A) across time to derive the corresponding raw trace during periods of rest and ambulation.

For ΔF/F measures, the Ca^2+^ signal was normalized to the fluorescence intensity at baseline (resting periods) and binarized to extract kinetic properties of individual events (Figure 3B, middle). For each aROI, an event was counted (> 2SD from mean of the raw Ca^2+^ trace at rest), only if longer than 1 second in duration. This event was then used to extract specific Ca^2+^ transient properties including rise time, decay time, amplitude, and area‐under‐the‐curve (AUC) measures (Figures 3E–H). Baseline activity was quantified at rest, and based on the number of events per min initiated during these periods (Figure 3D). Events that were initiated during ambulation were not counted. Pairwise correlations across all binarized traces (showing at least one event across a ∼5.5 min imaging window) in each FOV, were used to quantify positive correlation coefficients (CCs, Figure 3I) and functional connectivity, defined as the number of connected aROIs with CCs > 0.8 (Figure 3J).

For continuous wavelet transform (CWT) measures, a Morse‐based CWT for each aROI (Ca^2+^ trace) was used to extract the power of the signal across several frequencies (0.02–14 Hz). For each frequency bin, the number of connections and the corresponding distance between pairs of astrocytes in the FOV were derived from measures of rolling CCs (*t* ± 50 samples; ∼3.3 second range) calculated from their CWT power. CCs > 0.8 were used to define positive connections (Figure 4D). Correlograms show an increase in activated and correlated astrocytes compared to the resting state (Figure 4C,D). The corresponding binarized velocity data (i.e., ambulation status) were then introduced to segment these network parameters into resting (velocity < 20 mm/sec) and ambulating (velocity≥20 mm/sec) categories (Figure 4E,F,H,I) and averaged accordingly. Due to the very slow nature of in vivo astrocytic Ca^2+^ waves (∼ 5–10 seconds), and for ease of comparison across multiple factors, we averaged data on some of the outcome measures (Figure 4G,J–N) within the 0.05–0.25 Hz (4–20 seconds) range.

While these variables are based on CWT power extraction, we also binarized the power trace to calculate astrocytic functional activity (i.e., density), proportional activity ratio, and the time‐based synchronicity of the astrocyte network. For each frequency bin, extracted power (red trace; second panel from bottom of Figure 4B) was used to binarize and filter for the presence of an event (> 2SD from the mean power, blue line) during resting and ambulation periods (Figure 4B bottom trace). Functional activity was derived from the number of active astrocytes (at least one event detected) per mm^2^ (Figure 4K). The proportional activity ratio (Figure 4L) was calculated as the number of active astrocytes divided by the total number of aROIs in the FOV. Time‐based synchronicity was measured by calculating the proportion of time each aROI was synched to either the 0.05, or the 0.1 Hz frequency during ambulation. Both numbers were averaged across aROIs and FOV (Figure 4M). This number reflects on the percent of time Ca^2+^ events are synchronized to specific frequencies.

**FIGURE 5:**
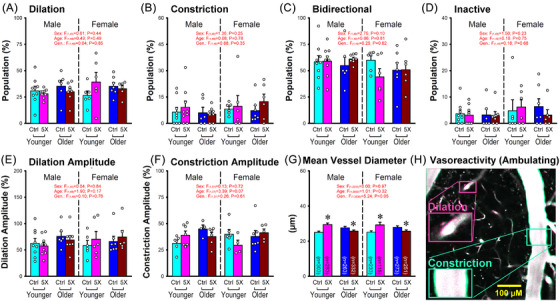
Vasoreactivity profiles and vessel properties. (A–D) Vessels were categorized to include dilation, constriction, both (bidirectional), or inactivity. No main effect was noted. (E, F) Analysis of dilation and constriction amplitudes also showed no main effects. (G) A significant main effect of genotype (Gen.) was seen on vessel diameters. (H) Representative photomicrograph of a field of view (FOV) showing constricting (green) and dilating (pink) vessel areas during ambulation. Error bars represent standard error of the mean.

Vascular ROIs (vROI) were defined using the average of the image cubes across time, binned into ∼1.33 second segments (40‐frame averaging). A Gaussian blurriness (sigma = 1.2), a 3D adaptive thresholding (sensitivity = 0.26), and a 2D size filter (> 160 µm^2) were used on each frame to identify vROIs. Only vROIs present from the beginning to the end of the imaging session (∼5.5 minutes) were used for measures of vasoreactivity. For each FOV, we compared the changes in vROI size (pixels) across periods of ambulation and rest to extract the proportion of vessels showing an increase > 2SD from the resting value (dilation, Figure 5A), a decrease > ‐2SD from the resting value (constriction, Figure 5B), a bidirectional response (crossing both thresholds, dilating and constricting, Figure 5C), or no change (staying within both thresholds, inactive, Figure 5D). Dilation amplitudes (Figure 5E) were derived from the dilation data presented in Figure 5A using the percent change from baseline for each vROI. Similar approaches were used for constriction amplitude changes (Figure 5F). Lastly, we also analyzed the vessel size distribution across all FOVs and groups. Diameter of each vROI (µm), irrespective of the imaging plane (i.e., penetrating, angled, parallel) was extracted by fitting a circle of maximum size within the lumen of each vessel.

### 2.7 Cross‐correlations between variables at the endfeet

**FIGURE 6:**
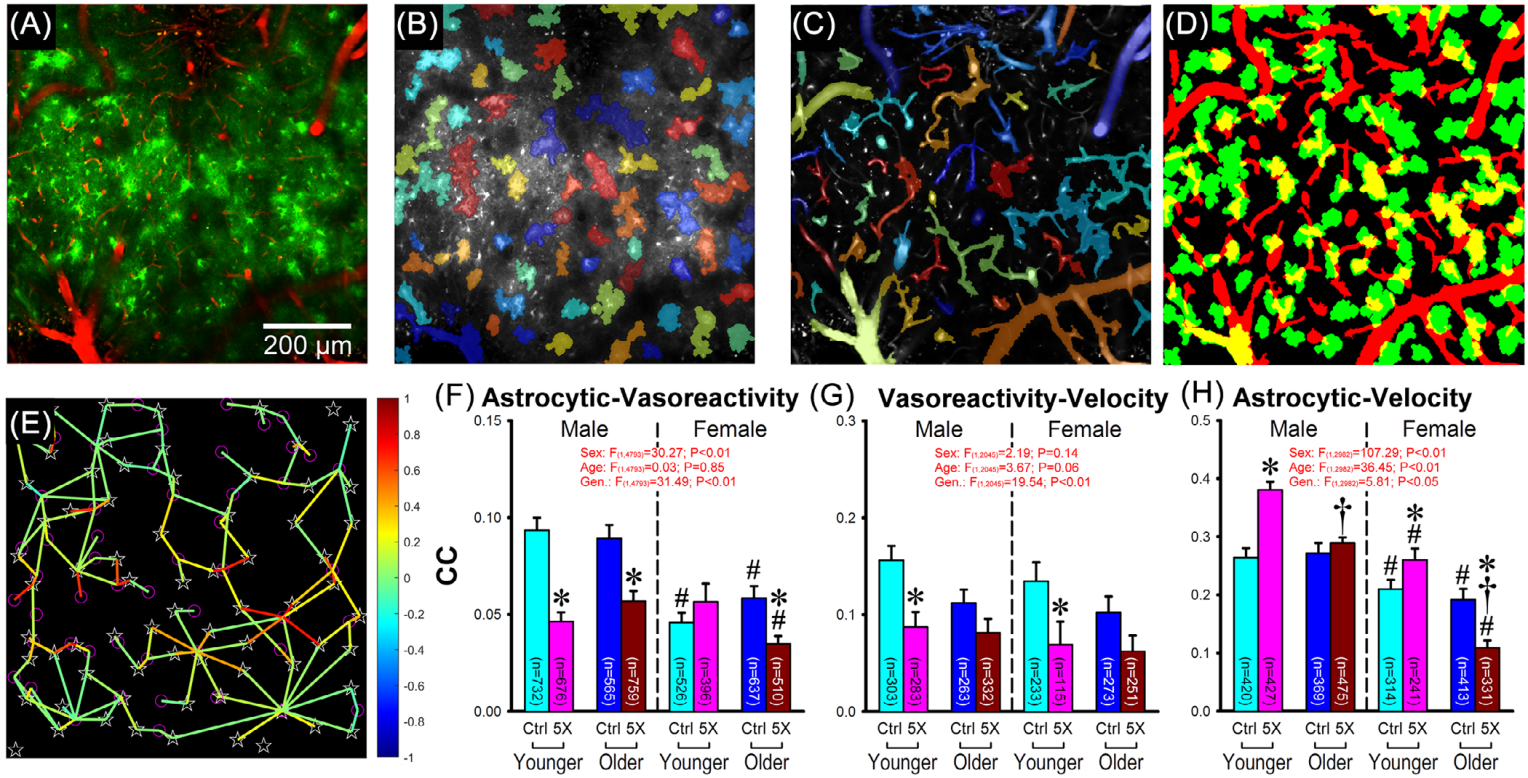
Vascular region of interest (vROI) identification and cross correlations between astrocytic Ca^2+^, vasoreactivity, and ambulation (velocity). (A) Representative image stack of astrocytic Ca^2+^ signals (green) and vasculature elements (red) used to identify astrocytic regions of interest (aROIs) (B) and vROIs (C). (D) vROIs and extended aROIs were used to identify overlapping areas (yellow) and to extract the endfeet Ca^2+^ trace for correlation with the neighboring vasoreactivity (F). (E) White stars (☆) represent centroid of each aROI; magenta circles (°) represent centroid of each vROIs; line color indicates the strength of the correlations (color bar, right) and shows heterogeneity in aROI responses as well as vROI activity in the FOV. (F) Correlation coefficients (CC) between astrocytic endfeet Ca^2+^ and local vasoreactivity. Main effects of sex and genotype (Gen.) were noted with greater reductions in female 5×FAD mice perhaps highlighting decoupling. (G) CC between vasoreactivity and ambulation (velocity). A main effect of genotype was noted, again, with reduction in the 5×FAD animals. (H) CC between astrocytic Ca^2+^ and velocity. Main effects of sex, age, and genotype were noted. * indicates significant difference from their corresponding controls (Ctrl); † indicates significant difference from the younger group; # indicates significant difference from the corresponding male group. Error bars represent standard error of the mean.

To calculate associations between vasoreactivity and other measures at the endfeet (astrocytic Ca^2+^ changes and velocity) we first resampled (using interpolation) the vROI traces (see 40‐frame average above) to match other variables’ sampling rate (30 Hz). Both measures of Ca^2+^ traces and velocity traces were, therefore, smoothed using a 2‐second rolling average. In order to maximize our ability to identify interacting domains between vessels and astrocytes either below or above the focal plane, for each FOV, we expanded individual aROIs by 15 µm in all directions to search for overlapping vROIs. This created an approximate neighborhood area corresponding to endfeet signals (yellow areas in Figure 6D). We extracted the Ca^2+^ signals from these overlapping regions across the imaging window (∼5.5 minutes; Figure 6E,F) and quantified the correlation with the local vasoreactivity. Correlations between vasoreactivity and ambulation were obtained across the imaging window (Figure 6G). A similar approach was used to measure correlations between an animal's ambulation and astrocytic Ca^2+^ signals (Figure 6H).

### 2.8 Velocity data extraction

The mouse ambulation velocity trace, extracted from the locomotion tracking software (Neurotar), was binarized using a threshold value greater than 20 mm/s to define periods of ambulation. Pauses between bouts of ambulation less than 3.3 seconds were ignored. This approach allowed us to identify periods of rest and ambulation across a ∼5.5 minute recording window. For measures of correlations between animal velocity and other measures, a 2‐second rolling average was used resulting in the same sampling rate on each variable (see the Cross‐correlations between variables at the endfeet section).

### 2.9 Statistics

Data are represented as means ± standard error. We used Prism (GraphPad software Inc. San Diego, CA) and SigmaPlot V14.0 (Systat software Inc., San Jose, CA) to test for significance across groups and report on main effects using three‐way analysis of variance (ANOVA). All CCs reported use Pearson's R measures. All data presented were tested for normality and passed the Kolmogorov‐Smirnov test.

## 3 RESULTS

### 3.1 Mice exhibit age‐related gait dysregulation

We tested for the presence of gait dysregulation in control and 5×FAD mice (*n* = 51) across age and sex using previously published outcome variables.^39^, ^40^ Earlier reports show that in other models of amyloidosis, gait alterations have been seen,^41^, ^42^ and here, the insulin receptor (IR)‐floxed 5×FAD mice also displayed altered ambulation (see Figures 1A and 1B). Surprisingly, this model did not display reductions in ambulation speed (Figure 1C), but did display age‐ (*F*_(1,43) _= 10.10, *p* < 0.01) and genotype‐dependent (*F*_(1,43) _= 34.46, *p* < 0.01) increases in the number of steps taken per cm (Figure 1D), perhaps mirroring gait dysregulation seen in the clinic with age or AD.^36^, ^37^, ^38^ We also noted age‐ (*F*_(1,43) _= 10.83, *p* < 0.01) and genotype‐dependent (*F*_(1,43) _= 37.54, *p* < 0.01) reductions in stride length deviance, perhaps highlighting a less confident ambulatory performance (Figure 1E). To address the animals’ ability to ambulate in a straight line, we measured deviance from center of the corridor for each step, and identified age‐dependent (*F*_(1,43) _= 19.04, *p* < 0.01) increases in this variable (Figure 1F). Given that these gait characteristics appear similar to those seen in patients early in AD, our results support the need for continued investigations in the somatosensory cortex where neuronal as well astrocytic network may contribute to the poor motor coordination seen in animal models of amyloidosis.

### 3.2 Elevated Aβ deposition in the IR‐floxed 5×FAD mouse

Because this genetically modified model of amyloidosis (IR‐floxed 5×FAD) was derived in‐house and no prior reports have quantified amyloid beta (Aβ) load in this animal model, we quantified plaques in S1 of animals across age and sex (*n* = 4 per group). As predicted, with increasing age, Aβ deposition increases significantly between 3 and 8 months of age (Figure 2A,C) only in the 5×FAD animals (*F*_(1,24) _= 45.52; *p* < 0.01). Indeed, a main effect of genotype was seen (*F*_(1,24) _= 60.94; *p* < 0.01) with no significant differences across sex (Figure 2C). It is of interest to note that using similar animal models (5×FAD crossed with an inducible IR knockout), Khan and colleagues have previously reported on enhanced Aβ accumulation in mice hippocampi with reduced astrocytic IRs.^43^ Here, the results on plaque load accumulation suggest the IRflox genetic manipulation did not interfere with the 5×FAD transgenes on the production of plaques. We investigated astrocyte reactivity in S1 and unmasked a significant genotype effect (*F*_(1,24) _= 10.39; *p* < 0.01) of increased green fluorescence intensity (GFAP), normalized to DAPI nuclear staining (Figure 2B,D). These results were anticipated and reflect on the relatively innocuous impact of inserting the IRflox transgene in the 5×FAD animal model.

### 3.3 Astrocytic Ca^2+^ event properties

We first used Δ*F*/*F* approaches on individual raw Ca^2+^ traces to extract events (> 2SD from mean at rest; Figure 3C) and measured astrocytic baseline activity at rest across all aROIs (Table 1). These results point to an age‐dependent increase in activity mostly in males (*F*_(1,2982) _= 50.24, *p* < 0.01), as well a main genotype effect with increased baseline activity in 5×FAD, compared to controls (*F*_(1,2982) _= 13.20, *p* < 0.01; Figure 3D). However, we also note that a significant sex‐dependent reductions in activity was seen, primarily in aged females (*F*_(1,2982) _= 8.38, *p* < 0.01). The next analysis pooled the individual astrocyte values across FOVs and shows a strong sex difference in measures of Ca^2+^ transient amplitudes (Figure 3G) and AUC (Figure 3H) with reductions in females compared to males. Other kinetic parameters measured included rise and decay time constants, and were not significantly different across age, sex, or genotype (Figure 3E,F). From the binarized data (Figure 3B middle panel), pairwise correlations across astrocytes (Figure 3I) revealed significant sex differences again, showing reductions in this variable in the female group. Strong sex‐dependent (*p* = 0.06; Figure 3J) as well as genotype‐dependent (*p* = 0.06; Figure 3J) trends were identified on measures of functional connectivity, again, with reductions in females and in 5×FAD animals. The reduction in functional connectivity and pairwise correlations align relatively well with a recent published report in the same 5×FAD model^30^ but were not anticipated, given the longer nature of astrocytic Ca^2+^ transients and the previously reported astrocytic hyperactivity phenotype (see the Introduction section). Indeed, we predicted that increased excitability and synchronicity in an astrocyte network would increase pairwise correlations.

### 3.4 Astrocyte Ca^2+^ network properties

We used Morse CWT to address the strength of the Ca^2+^ signals (i.e., power) across multiple encoding frequencies (0.02–14 Hz) in individual aROIs from each FOV (Figure 4A) as previously described^39^, ^40^. Briefly, CWT‐extracted intensity traces from each aROI were used to derive the power across frequencies and time (see methods). Examples of CWT‐extracted (0.1–0.25 Hz) alterations in CCs and functional connectivity are shown as correlograms in Figures 4C (resting) and D (ambulating). Not surprisingly, given the slow nature of Ca^2+^ waves and extensive direct gap junction interconnectivity in astrocytes, a clear and significant main effect of frequency was noted on all extracted variables (Figure 4E,F,H,I) highlighting CCs approaching 1, together with increased connectivity at lower frequencies.

Pairwise positive correlations were averaged to yield a mean paired CC for each FOV (Figure 4E–G) and are presented across the frequency domains, genotype, age, and sex at rest (Figure 4E), and during ambulation (Figure 4F). To downsize the number of factors presented (frequency, age, sex, ambulation status, and genotype), we reduced the frequency domain to the 0.05–0.25 Hz range, and calculated the CC differences between resting and ambulating. Using this approach, the change in these CCs (difference between resting and ambulating) is presented in Figure 4G and highlights a significant main effect of sex (*F*_(1,49) _= 13.61; *p* < 0.01) with reduced CCs in females compared to males, along with a trend for reduced CCs across genotype (*F*_(1,49) _= 3.46; *p* = 0.07). Note that these results align with Figure 3I.

Functional connectivity measures based on pairwise CCs > 0.8 taken at rest and during ambulation also reveal a main effect of age in both sexes (Figure 4H,I). Interestingly, in C57BL6J, we have previously noted an age‐dependent increase in neuronal network synchronicity using similar approaches, but only in older males.^39^ Further, older F344 male rats also show increases in network connectivity and overall activity^40^ suggesting that age‐dependent processes do not recapitulate those controlling network activity in models of amyloidosis. Here, we do note a significant genotype effect in ambulating females (Figure 4I, right), this time, displaying reduced connectivity (*F*_(1,252) _= 4.08; *p* < 0.05). We also measured the differences in CCs and functional connectivity between rest and ambulation in order to reduce the complexity of the analysis and focused on the 0.05–0.25 Hz activity range (Figure 4G,J). Both results again yielded a significant main effect of sex, with reductions seen in females for ΔCC (*F*_(1,49) _= 13.61; *p* < 0.01) and for Δ functional connectivity (*F*_(1,49) _= 11.62; *p* < 0.01). A main genotype effect, highlighting reductions in the 5×FAD group, was also revealed for Δ functional connectivity (*F*_(1,49) _= 4.48; *p* < 0.05).

We also used this CWT‐based approach to quantify astrocytic network variables (Figure 4K–N). Functional activity (number of active astrocytes/mm^2^) and the proportional activity ratio (active astrocytes/ total number of of astrocytes) per FOV were not significantly altered, suggesting astrocyte density was not changed across groups (Figure 4K,L, respectively). We found that the astrocytic network synchronicity was reduced in the 5×FAD animals compared to controls (*F*_(1,49) _= 14.34; *p* < 0.01; Figure 4M), with greater reductions in females (*F*_(1,49) _= 14.05; *p* < 0.01; Figure 4M). Of note, a main effect of age was seen on measures of distance between active astrocytes (*F*_(1,49) _= 8.67; *p* < 0.01), with a small, but significant, increase in the aged groups (Figure 4N).

### 3.5 Vasoreactivity

For each FOV we investigated vasoreactivity from the changes in rhodamine dextran fluorescence, by calculating the variation in size for each vROI across time (∼5.5 min). We quantified the proportion of vROIs that either dilated (Figure 5A), constricted (Figure 5B), responded bidirectionally (Figure 5C), or stayed inactive (Figure 5D). We also measured the amplitude of the dilation (Figure 5E) and the constriction (Figure 5F) for each vROI (see the Methods section), as well as the distribution of vessel diameters across groups (Figure 5G). None of these measured showed main effects of age, sex, or genotype. A trend was noted; however, for an aging effect in males and females, reporting on increases in constriction amplitudes when compared to younger animals (Figure 5F). This result aligns relatively well with the decrease in dilation seen in the same animal model albeit at slightly younger ages.^30^ Here we investigated measures of vasoreactivity across entire FOVs irrespective of vessel size or depth (∼40–400 µm), but excluded large pia arteries. A sub‐analysis of the vROI dataset investigated vessel sizes and shows that most vessels ranged from 10 to 60 µm in diameter and had very similar size representation amongst the groups of animals (Figure S1).

### 3.6 Correlations between variables

We used an unbiased approach across full FOVs to investigate local neighborhood correlations between vROI and aROIs and focus our analysis on the physical overlap between each ROI (i.e., endfeet, see the Methods section). The relationship between vasoreactivity (dilation, constriction, bidirectional, inactive, see Figure 5A–D) and regional astrocyte Ca^2+^ changes at the endfeet highlights main effects of sex (*F*_(1,4793) _= 30.27; *p* < 0.01) and genotype (*F*_(1,4793) _= 31.49; *p* < 0.01) with decreased CCs in females compared to male, along with reductions in 5×FAD compared to controls (Figures 6F). Clearly, older 5×FAD animals show very robust reductions in astrovascular coupling (decreased ∼35%–40%). Further, an analysis of the relationship between vasoreactivity and ambulation status (velocity) also reflects the presence a main genotype effect (*F*_(1,2045) _= 19.54; *p* < 0.01; Figure 6G). 5×FAD animals, again display reduced correlations between ambulation and vasoreactivity, suggesting vessel reactivity is decoupled. Finally, correlations between astrocytic Ca^2+^ changes and ambulation also unmask significant reductions in these values across age, sex, and genotype (Figure 6H). Again, older female 5×FAD mice display the most robust dysregulated phenotype compared to other groups, reflected by severe decoupling between astrocytic Ca^2+^ signals and ambulation.

## 4 DISCUSSION

Here we report, for the first time, on astrocytic network measures at rest and during ambulation across age, sex and genotype in 57 animals. Using an unbiased analysis approach across whole FOV containing nearly 3000 individual astrocytes and 2000 vascular elements (Table 1), clear reductions in the coupling between astrocytes and vasoreactivity were identified. We emphasize the neutral nature of the approach without attention to specific areas within a FOV that are more or less reactive to movement, therefore including all vessels and astrocyte Ca^2+^ network analysis reveals significant reductions in measures of astrocytic communication and activity, and were associated with reduced functional vasoreactivity in awake ambulating animals. We also suggest the S1 astrocytic network alterations may contribute to central mechanisms that reflect on the associations between AD and gait dysregulation.

### 4.1 Gait

In both large European and North American cohorts, negative associations between ambulation pace and dementia have been reported.^44^, ^45^ Worldwide data highlight evidence that central alterations within at least two brain regions controlling very different modalities (memory and ambulation) may share common dysregulation pathways. In support of this idea, it has become clear that when dual cognitive and mobility decline coincide, a heightened risk of dementia is seen,^38^, ^46^, ^47^, ^48^ suggesting that a central mechanism across brain regions could underlie gait and cognitive decline in AD. For these reasons, we chose to characterize gait parameters using clinically‐relevant variables previously published by our group, while investigating Ca^2+^ network dynamics between astrocytes as well as between astrocytes and the vasculature in freely ambulating mice, with a focus on the somatosensory cortex.

Across a cohort of 51 animals, we found alterations in gait, evidenced by main effects of age and genotype, where older 5×FAD animals display shorter stride lengths (Figure 1E). These results align with previous work in this and other animal models of amyloidosis that have reported ambulatory/gait dysregulation.^41^, ^42^ Similar phenotypes in the 5×FAD mirror elements of gait dysregulation seen in the clinic, including increases in double support time, shuffling gait, and reduced stride length.^36^, ^37^, ^38^ Our measures of association between bouts of ambulation and astrocytic Ca^2+^ in S1 (Figures 4M and 6H) highlight clear reductions in network synchronicity and correlations, particularly in older 5×FAD females, perhaps indicative of reduced sensory modalities or encoding within S1. Thus, the central sensory alterations uncovered here may shed new light onto the potential mechanisms linking gait dysregulation in AD. These results, of course, do not preclude the contributions of elements associated with motor performance and movement execution, which are also known to be impaired with advanced age and/or AD.^49^

### 4.2 Calcium transients

Astrocytes Ca^2+^ transients have been hypothesized to encode changes in general arousal (i.e., heightened vigilance) during locomotion, but also to coordinate neuronal networks through synchronized norepinephrine (NE) signals. Indeed, adrenergic blockers confirm the role of cortical astrocytic Ca^2+^ in awake, behaving mice during locomotion as well as during startle responses.^50^, ^51^, ^52^, ^53^ Further, during ambulation, reductions in astrocytic Ca^2+^ networks, driven by reduced cortical NE levels, have been identified in plaque‐bearing mice.^27^ Importantly, and irrespective of the underlying mechanisms responsible for the Ca^2+^ transients measured here at rest and during ambulation (ΔF/F), we find reductions in the amplitudes of the Ca^2+^ signals in females compared to males (Figure 3G) along with reductions in the AUC (Figure 3H) and astrocytic pairwise correlation coefficients (Figure 3I), results that partially align with a recent report in another transgenic model of amyloidosis where reactive astrogliosis is pervasive.^54^

Thus, reduced astrocytic communication and functional connectivity (Figure 3J, sex trend) could be responsible for the noted decoupling between astrocytes and vasoreactivity (Figure 6F,H) reported here in females. Whether this is based on reductions in the effectiveness of the communication or desynchronized activity (i.e., increasing noise) remains to be determined. What is important to note here is that a reduction in Ca^2+^, rather than an increase, appears to be driving this phenotype in the older 5×FAD females. This may appear controversial, but others have suggested that, perhaps due to temporal dissociation between the two signals, astrocytic Ca^2+^ transients may be disconnected from hyperemic control.^23^, ^24^, ^25^, ^26^, ^30^ As mentioned, decreased Ca^2+^ signaling or sensory‐evoked responses were found in the APP NL‐F model,^13^ the Tg‐Arc/Swe mouse,^27^ the APP/PS1,^19^, ^28^ and the 5×FAD mouse.^29^ Together, therefore, we find reduced synchronization and functional connectivity in the astrocyte network is associated with astrovascular decoupling.^30^

We did note the presence of increased astrocytic baseline activity at rest in older males compared to younger males, as well as in 5×FAD compared to controls (Figure 3D), perhaps aligning with prior work showing astrocytic hyperactivity and dysregulation of Ca^2+^‐dependent signaling.^55^, ^56^ In principle, this increase in baseline activity could help explain the reductions in functional connectivity (Figure 4J) and synchronicity (Figure 4M) seen here, whereby the *uncoordinated* activity could decrease network synchronicity; however, this is unlikely for several reasons. First, older males do not show increases in baseline activity (5×FAD compared to control) but show significant reductions in functional connectivity, time‐based synchronicity, and astrovascular correlations (Figures 3, 4, and 6F). Second, the baseline activity is very low (∼1 event per 5 min, 0.0033 Hz) and is therefore unlikely to contribute to reductions in these network properties in 5×FAD compared to controls. Third, females reliably display greater alterations in nearly all network variables reported here, yet show reduced baseline astrocytic activity when compared to males (Figure 3D). Fourth, mean paired CC measures at rest do not show significant genotype differences across groups (Figure 4E), even though the older males, irrespective of genotype, display the highest baseline activity measures (Figure 3D).

### 4.3 Vasoreactivity

Categorizing vascular changes (dilation, constriction, bidirectional, or inactive, Figure 5A–D), we find no significant changes across the four groups of animals tested. Measures based on the amplitude of the dilation changes whether constricting or dilating also did not change across the groups (Figure 5E and F). Measures of vessel characterization across > 2000 vROIs note a small, albeit significant increase in diameters in the 5×FAD groups irrespective of sex (Table 1 and Figure 5G). Surprisingly, in older animals this relationship flips, where both sexes report on a significant decrease in vessel diameter in the 5×FAD compared to controls. Whether these small decreases in average diameter in the 5×FAD older animals align with the well‐recognized clinical manifestation of the disease on measures of cerebral blood flow and blood‐oxygenation‐level‐dependent imaging remains controversial, but it is likely to be very impactful. This is because of the inverse relationship between resistance and vessel diameter changes (including the fourth power of the radius change [Poiseuille's law]) when considering blood flow changes. As an example, the ∼9% reduction in vessel diameter in older 5×FAD females corresponds to a ∼40% increase in vascular resistance. However, we find that changes in vessel sizes imaged here are not sensitive to sex and, thus, not likely to reflect on the significant impact of the vasoreactivity dysregulation highlighted here in the older 5×FAD females.

A more focused analysis investigating the relationships between astrocytic Ca^2+^ endfeet and vasoreactivity changes across animal groups revealed significant main effects of sex and genotype (Figure 6F) highlighting reductions in both cases on astrovascular coupling and possibly, decoupling between the two factors.^30^ This change persisted outside of the endfoot region; however, as evidenced by reductions in measures of ambulation‐driven vasoreactivity (Figure 6G), and ambulation‐driven astrocytic Ca^2+^ (Figure 6H). Together, these results again suggest Ca^2+^ reductions may be driving vascular decoupling irrespective of the astrocytic region tested.

### 4.4 Overall

Our work aligns well with prior evidence showing disconnections between astrocytic Ca^2+^ and vascular control.^23^, ^24^, ^25^, ^26^, ^30^ Importantly, we also show that sex and/or age are clear covariates that contribute to the phenotypes seen in this animal model of amyloidosis. However, it is important to note that, while females are clearly more impacted than males, the relationships with Aβ deposition are more ambiguous. Indeed, prior to any significant plaque deposition, young 5×FAD males display enhanced baseline activity and dissociation between astrocyte Ca^2+^ responses and vasoreactivity (Figures 3D, and 6F). This could be due to oligomeric forms of Aβ that exist prior to plaque formation and may influence astrovascular coupling. Indeed, work from several groups, including analyses of the impact of Tau oligomers, has presented evidence for significant alterations in astrocytic calcium homeostasis.^20^, ^57^ It is also difficult to reconcile notions of network hyperexcitability with negative downstream consequences on astrovascular coupling (i.e., decoupling). Here, we posit that reductions in astrocyte network communication variables and Ca^2+^ transients reduce functional coupling with the vasculature in this model of amyloidosis. Ultimately, the reduction in Ca^2+^ signal amplitudes in older female astrocytes could result in reductions in event detection reliability (beyond endfeet domains), causing reductions in baseline activity, positive astrocytic CCs, and functional connectivity measures. A caveat that needs consideration is that, while we describe main effects of aging, we use the term to encompass a period of aggressive amyloidosis, from early deposition (3–4 months), to more established and severe pathology (8–10 months) in contrast to traditional aging studies that often use much older animals (e.g., 22–36 months). The model of amyloidosis used here does not permit for long‐term aging studies into such advanced ages.

By design, our investigation was strictly associative and linked important variables known to control vasoreactivity and thus, it is difficult to infer on the underlying mechanisms that contribute to the very large sex‐dependent effects seen. Here, we suggest that the lack of vasoreactivity orchestration may stem from the dysfunctional control of vessel tone via reductions in astrocytic Ca^2+^ signal strength, connectivity, correlation coefficients, and synchronization. Given the lack of vessel reactivity changes, and the small, yet likely impactful changes in vessel morphology (Figure 5), we suggest the impaired astrocytic Ca^2+^ network properties identified here, may be responsible for the astrovascular decoupling.

## CONFLICT OF INTEREST STATEMENT

The authors declare no conflicts of interest.

## CONSENT STATEMENT

No human data were derived and, therefore, consent was not necessary.

## Supporting information



Supporting Information
