# Supplementary material for: Astrovascular decoupling in awake 5×FAD mice is associated with reduced astrocytic calcium
Source: Alzheimers Dement. 2025 Aug 13;21(8):e70564. doi: 10.1002/alz.70564 (PMC12344447; doi:10.1002/alz.70564)
Supplement: Supplementary file 1 — Supporting Information [file ALZ-21-e70564-s001.pdf]

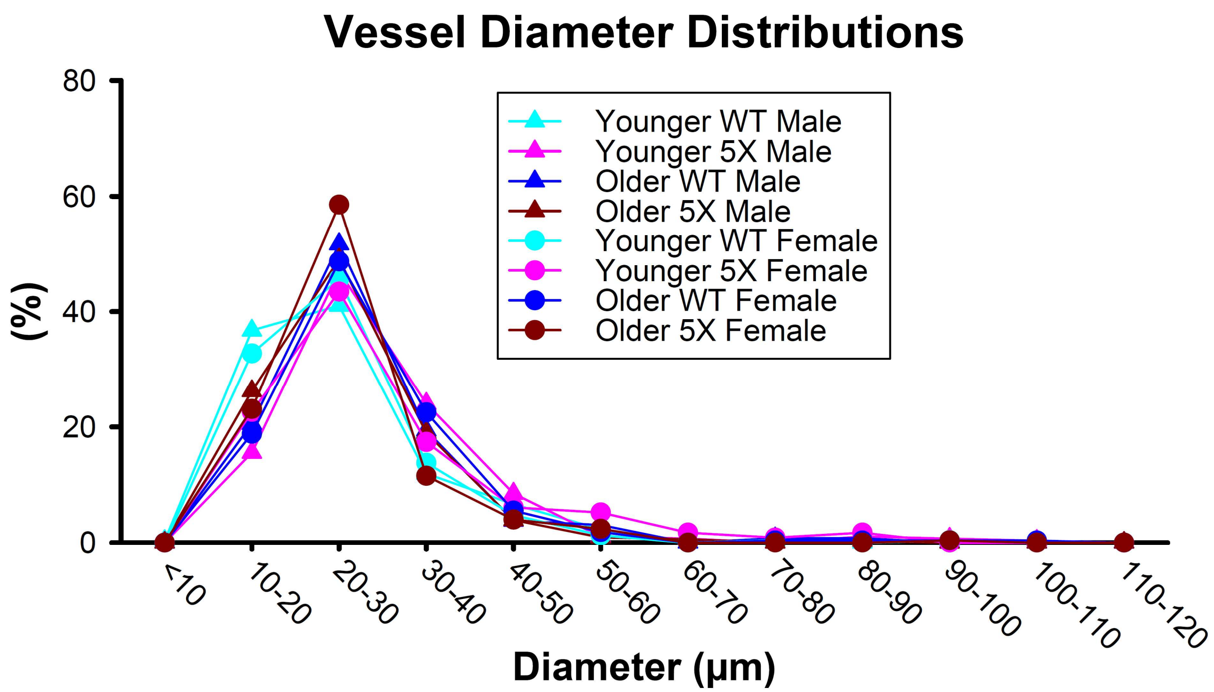

Supplemental Figure 1. **Vessel diameter distributions across groups.** For each animal group, we tabulated the proportional representation (%) of vROIs across vessel diameters imaged. No clear size overrepresentation in one group, or shift in the vROI distribution were noted.
